# Effects of Pilates Exercise Programs in People With Chronic Low Back Pain: A Systematic Review

**DOI:** 10.1097/MD.0000000000000383

**Published:** 2015-01-30

**Authors:** Antonino Patti, Antonino Bianco, Antonio Paoli, Giuseppe Messina, Maria Alessandra Montalto, Marianna Bellafiore, Giuseppe Battaglia, Angelo Iovane, Antonio Palma

**Affiliations:** Sport and Exercise Sciences Research Unit (A Patti, AB, GM, MAM, MB, GB, AI, A Palma), University of Palermo; Posturalab (A Patti, GM), Italy; and Department of Biomedical Science (A Paoli), University of Padua, Padua, Italy.

## Abstract

The Pilates method has recently become a fast-growing popular way of exercise recommended for healthy individuals and those engaged in rehabilitation. Several published studies have examined the effects of Pilates method in people with chronic low back pain (LBP).

The objective of this study is to describe and provide an extensive overview of the scientific literature comparing the effectiveness of the Pilates method on pain and disability in patients with chronic nonspecific LBP. The study is based on the data from the following sources: MEDLINE-NLM, MEDLINE-EBSCO, Scopus Elsevier, Cochrane, DOAJ, SciELO, and PLOSONE.

Original articles and systematic reviews of adults with chronic nonspecific LBP that evaluated pain and/or disability were included in this study; studies in which the primary treatment was based on Pilates method exercises compared with no treatment, minimal intervention, other types of intervention, or other types of exercises.

The Preferred Reporting Items for Systematic Reviews and Meta-Analyses (PRISMA) were adopted. The literature search included 7 electronic databases and the reference list of relevant systematic reviews and original articles to July 2014. Two independent investigators conducted the literature search and performed the synthesis as follows: Study Design; Sample (n); Disability measure; Intervention; and Main results.

The searches identified a total of 128 articles. From these, 29 were considered eligible and were included in the analysis. The items were stratified as follows: Pilates method versus other kind of exercises (n = 6 trials) and Pilates method versus no treatment group or minimal intervention for short-term pain (n = 9 trials); the therapeutic effect of the Pilates method in randomized cohorts (n = 5); and analysis of reviews (n = 9).

We found that there is a dearth of studies that clearly demonstrates the efficacy of a specific Pilates exercise program over another in the treatment of chronic pain. However, the consensus in the field suggests that Pilates method is more effective than minimal physical exercise intervention in reducing pain. These conclusions need to be supported by other proper investigations.

## INTRODUCTION

Musculoskeletal conditions (MSCs) are the most common cause of severe long-term pain and physical disability; in Europe, from 20% to 30% of adults are affected by MSCs at least once in their life.^1,2^ The burden that MSCs create has been recognized by the United Nations and World Health Organization, with their endorsement of the Bone and Joint Decade from 2000 to 2010.^3^ The prevalence of many of these conditions markedly increases with age and many patients also have some common lifestyle issues (obesity, smoking, and physical inactivity). With the increasing number of older people and the ongoing changes in lifestyle, the burden of MSCs and other noncommunicable diseases is predicted to increase.^4^ The Pilates method has recently become a fast-growing popular form of exercise recommended for healthy individuals and those engaged in rehabilitation. In more details, Pilates method emphasize core strengthening, posture, and coordination of breathing with movement, combining Asian and Western techniques.^5^ In 2009, Altan et al^6^ showed the effects of Pilates method in 49 women with fibromyalgia (ages 24–63 years). They demonstrated improvements in pain visual analog scale (VAS) scores. However, after an additional 12 weeks of follow-up, there were no differences between the groups. In 2001, Tuzer et al^7^ investigated if the psychological symptoms and the types of causal attributions were linked to the symptoms among women with chronic low back pain (CLBP). The authors showed that there was no difference between the groups regarding causal attributions. In this context, low back pain (LBP) is defined as pain and discomfort, localized below the costal margin and above the inferior gluteal folds, with or without leg pain. Nonspecific (common) LBP is defined as LBP not attributed to recognizable, known specific pathology. Pain cannot be attributed to pathology or neurological encroachment in about 85% of people.^8^ A role of genetic influence on liability to back pain is suggested from recent research. Hestbaek et al^9,10^ showed that LBP is usually dealt with as a specific and independent entity but the existing literature shows comorbidity to be common with LBP, suggesting that LBP may be part of a broader pattern of general health.^9–11^ Several published studies have examined the effectiveness of Pilates method in people with CLBP and reduction in pain when applying the Pilates method in treating nonspecific CLBP in adults.^12–14^ The literature defined Pilates method as a mind–body exercise that focuses on core stability, muscle control, breathing, strength, flexibility, and posture.^15^ This method (and the apparatus used during therapy sessions) was developed by Joseph Pilates. Pilates method involves conscious use of trunk muscles to stabilize the pelvic–lumbar region.^16^ These exercises can be performed using specific equipment (equipment-based Pilates) or without specific equipment (also known as mat Pilates). In 2014, Hyun et al^17^ showed that Pilates mat exercise was safer than exercise on an unstable base of support, and, in particular, the Pilates mat exercise program was easier to adjust to each individual's balance ability. These exercises aim to improve static and dynamic stability, as well as posture and movements in general.^15^ The objective of this study sounds is to describe and provide an extensive overview of the scientific literature comparing the effectiveness of the Pilates method on pain and disability in patients with chronic nonspecific LBP.

## MATERIALS AND METHODS

The Ethics Committee of the Sport and Exercise Sciences Research Unit, University of Palermo, Palermo, Italy, approved the systematic review in November 2013. The literature search was considered in line with ethical principles for medical research involving human subjects.

### Eligibility Criteria

Original articles and systematic reviews including adults with chronic nonspecific LBP that evaluated pain and/or disability; studies in which the primary treatment was based on Pilates method exercises compared with no treatment, minimal intervention, other types of intervention, or other types of exercises.

### Information Sources

Publications were selected based on a literature search from 2000 to 2014. The following databases were interrogated: MEDLINE-NLM and MEDLINE-EBSCO. We also searched on Scopus Elsevier, Cochrane, DOAJ, SciELO, and PLOSONE databases.

### Search Strategy and Keywords

The standardized search strategy included the use of the terms “Pilates” and “Low Back Pain” in the title, abstract, and keyword field. Preliminary searches have shown that expanding the search to include other keywords such as “exercise,” “motor control,” “core,” or the removal of “Low” in “Back Pain” did not identify any additional studies.

### Data Collection Process

All the retrieved articles were transferred into the Endnote software (Vers X6 for Windows 7, © Thomson Reuters). In the first stage, all findings were coded into 2 different categories: Reviews and Meta-analyses, and Original articles. This kind of codification was applied for each database interrogated. Afterward, we proceeded with the exclusion of all duplicates. We then proceeded with a qualitative synthesis of the full texts of the studies included. Two independent investigators conducted the literature search and performed the synthesis as follows: Study design; Sample (n); Disability measure; Intervention; and Main results. In cases of disagreement between the reviewers, a third reviewer was consulted to achieve consensus. To be considered in this systematic review, the below points were required:

1. Published in the English language, as access to interpreters was not available.
2. Published in full so that the methodological quality of the study could be assessed alongside results. Abstracts were excluded as they contained insufficient data to enable analysis of methodological quality.^18^
3. Assessed for the effectiveness of Pilates method where the term “Pilates” was used to describe the type of prescribed exercise being investigated. Exercises described as “motor control” or “lumbar stabilization” did not suffice for Pilates method. This is because Pilates method may include other features apart from motor control and lumbar stabilization.^15^
4. Included participants with CLBP, that is, localized pain in the lumbar region of >3 months in duration. If studies only included participants with LBP of <3 months duration, they were excluded. This is because people with CLBP respond differently to treatment compared to those with acute or subacute symptoms.^19^ If studies included participants with acute or subacute LBP and CLBP, the study was included as findings that were still considered relevant.
5. Used outcome measures with appropriate psychometric qualities that evaluate pain and/or functional ability in people with CLBP (the VAS, numerical rating pain scale [NRPS], the Oswestry disability questionnaire,^20,21^ Roland-Morris disability questionnaire,^21^ Borg scale CR10,^22^ Quebec back pain disability scale,^23^ patient-specific functional scale,^24^ pain self-efficacy questionnaire,^25^ and pain catastrophizing scale.^26,27^ Randomized controlled trial with outcome measures for pain and/or functional ability that did not have sufficient validity, reliability, or responsiveness were excluded to avoid not appropriate measurements of treatment effect.^27^

## RESULTS

**FIGURE 1:**
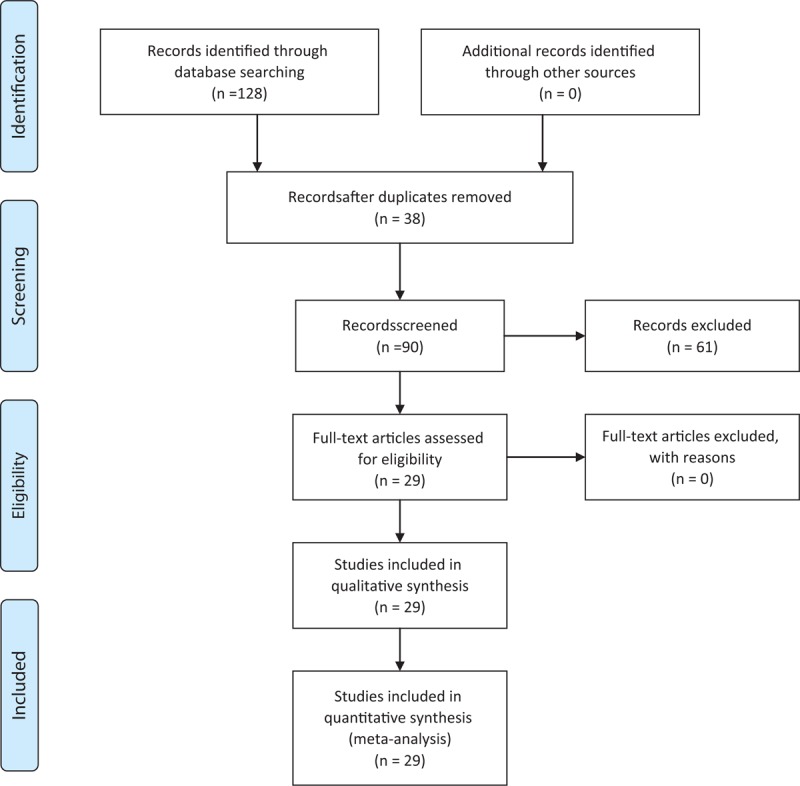
Flow of study.^28^

A total of 128 records have been identified through the database search of which 67 were considered potentially relevant and respected the previously mentioned inclusion criteria. Out of these, 38 articles were removed as duplicates, and we obtained 29 eligible articles (Figure 1 and Table 1  ).

**Table 1:**
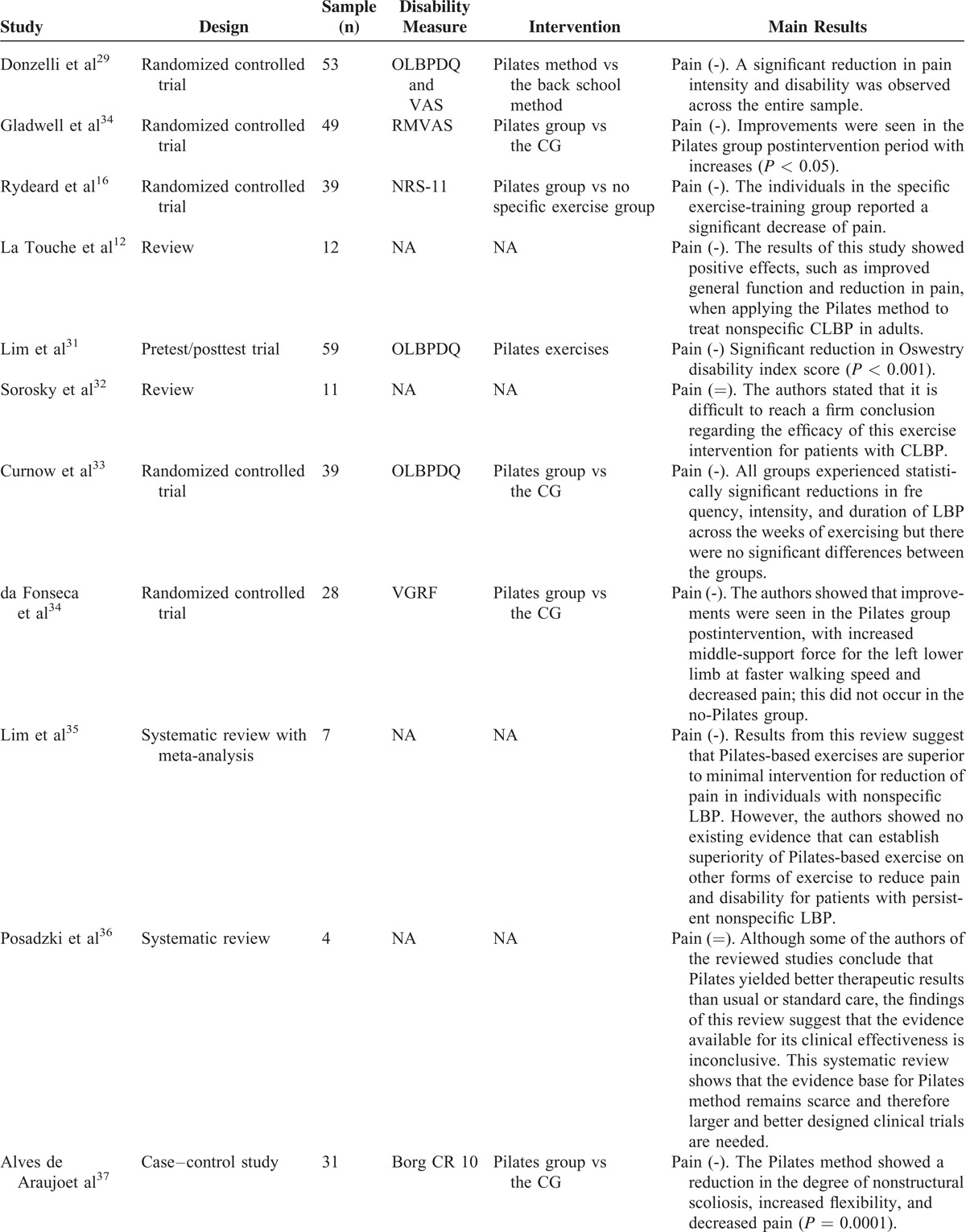
Overview of the Retrieved Reviews and Meta-Analyses

**Table 1 (Continued):**
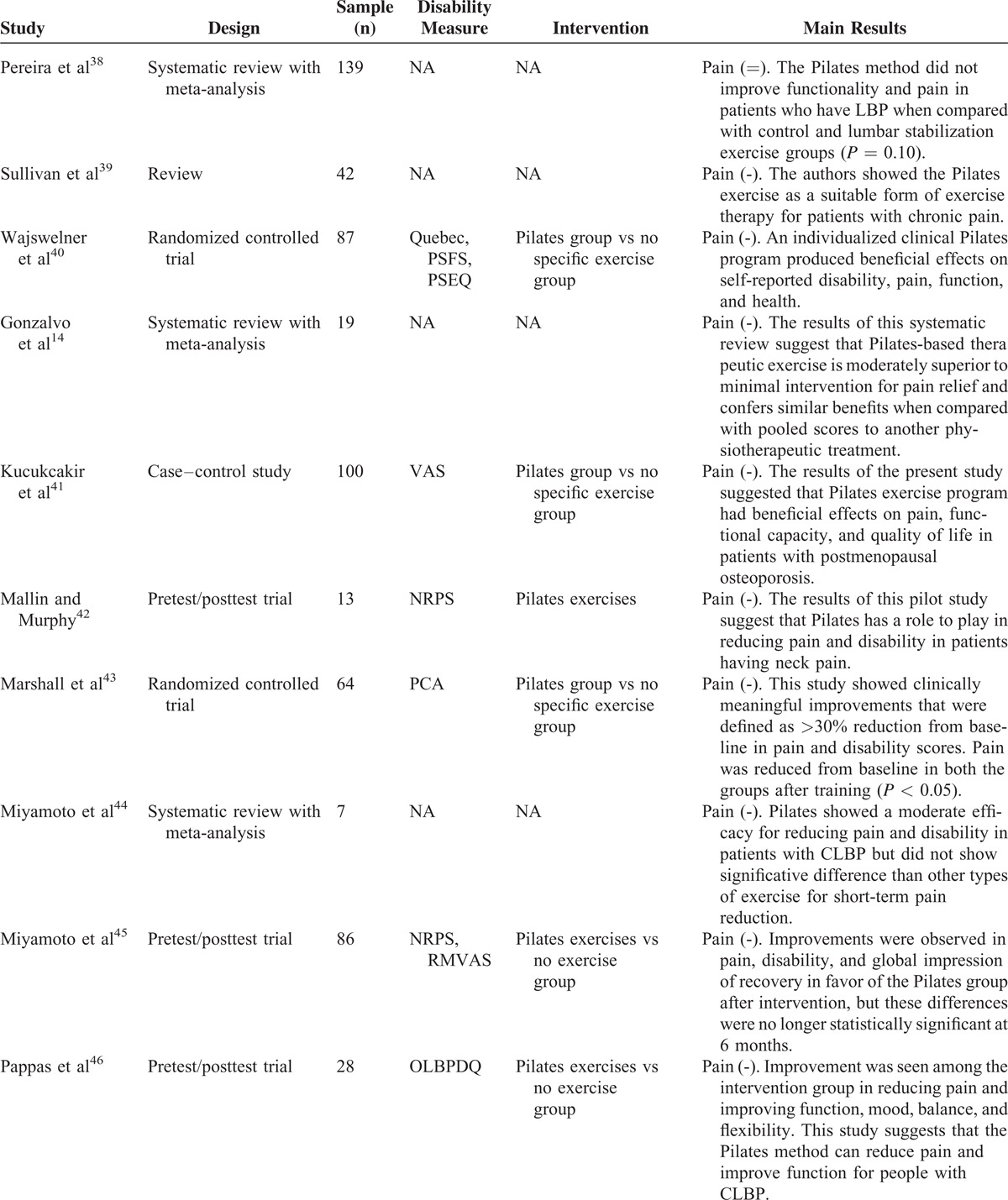
Overview of the Retrieved Reviews and Meta-Analyses

**Table 1 (Continued):**
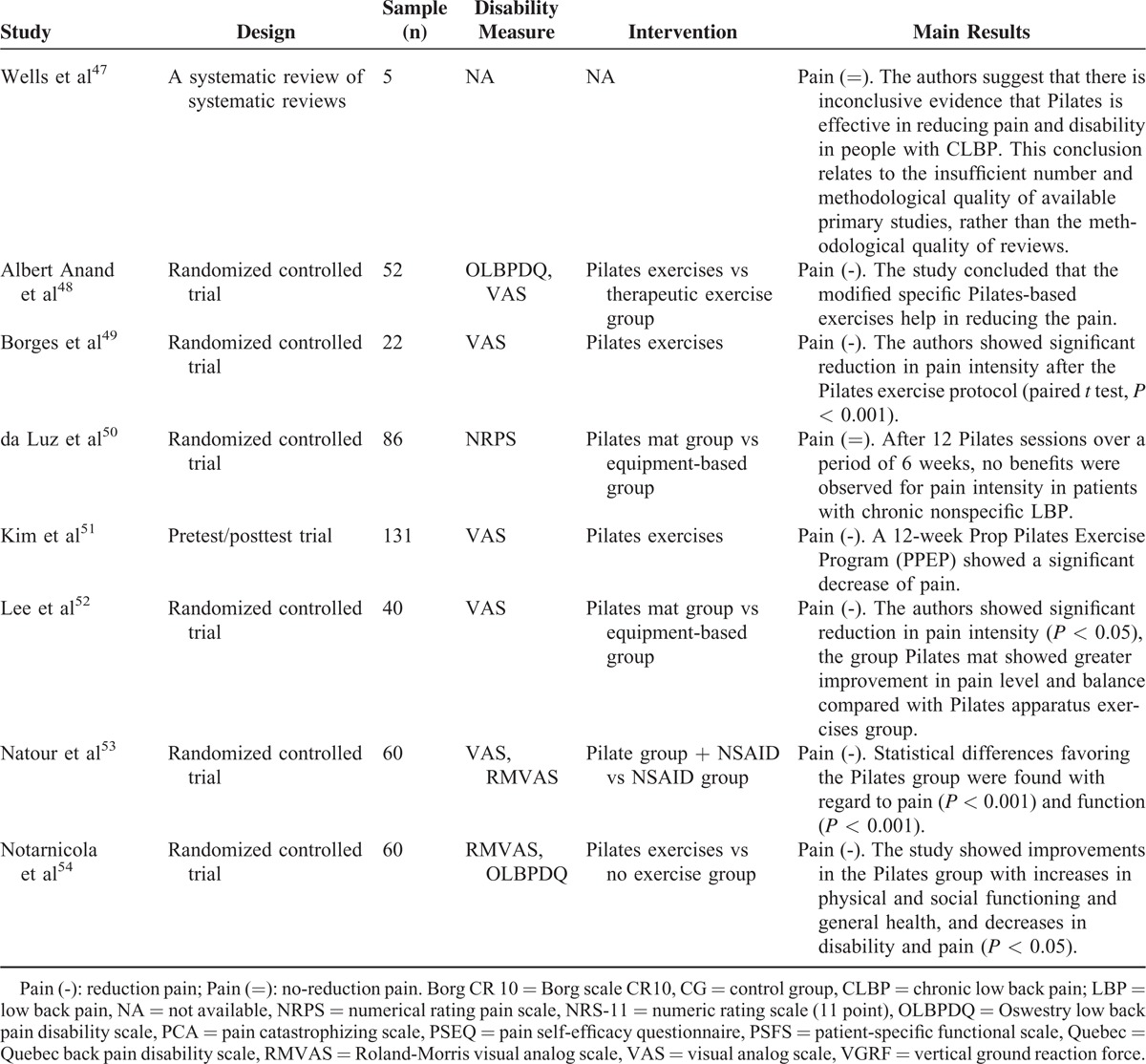
Overview of the Retrieved Reviews and Meta-Analyses

### Original Articles

This study included 21 randomized controlled trials; 20 studies showed a reduction of LBP but it was not possible to analyze the influence of the type of exercises on the analyses because the descriptions of the exercises performed in eligible studies were very brief.

#### Comparison of the Pilates Method With Minimal Intervention for Pain Outcome

Nine studies^16,30,34,37,45,46,48,53,54^ evaluated the pain before and after interventions and the results were compared with control groups (CGs) both with and without interventions and also with alternative pharmaceutical interventions. The study by Rydeard et al^16^ administered an exercise protocol with Pilates method-based floor exercises that was initially performed as static exercises protocol; they progressed to dynamic exercises involving hip extension movements and then to exercises on the reformer, with 12 one-hour sessions conducted in addition to a home-based program of 15 minutes, 6 days a week for 4 weeks. The individuals from the experimental group (EG) reported a significant decrease in LBP and disability, which was maintained over a 12-month follow-up period. Gladwell et al^30^ compared the Pilates method with a CG that continued with normal activity. The study showed a significant decrease in pain (*P* < 0.05), mostly, during the postintervention period. The authors suggest that Pilates method used as a specific core stability exercise incorporating functional movements can improve nonspecific CLBP reduction in active populations. Similarly, da Fonseca et al^34^ and Alves de Araujo et al^37^ compared the Pilates method with no exercise. In the study of da Fonseca, the Pilates group undertook 15 sessions of Pilates, and the data were collected before and after the intervention. After intervention, the Pilates group showed a significant decrease in pain and this did not occur in the no-Pilates group. In the study of Alves de Araujo, 31 female students, with scoliosis, were divided into 2 groups: a CG = 11, which had no therapeutic intervention, and an EG = 20, which underwent Pilates-based therapy. After intervention, the Pilates group showed a significant decrease in pain (*P* < 0.0001). The study by Miyamoto et al^45^ compared Pilates method treatment with giving patients an educational booklet (n = 86). The Pilates method was administered for 12 one-hour sessions over 6 weeks. Improvements were observed in pain (mean difference = 2.2 points, 95% confidence interval [CI] = 1.1 to 3.2), disability (mean difference = 2.7 points, 95% CI = 1.0 to 4.4), and global impression of recovery (mean difference = −1.5 points, 95% CI = −2.6 to −0.4) in favor of the Pilates group after intervention, but these differences were no longer statistically significant at 6 months. A study by Pappas et al^46^ showed results that suggest that as the Pilates method can reduce pain and improve function for people with CLBP, in comparison to no intervention. The study enrolled 28 patients, aged 20–60 years, with CLBP divided into 2 equal groups, an exercise group and a CG. The intervention group followed a Pilates method program with fitball for 6 weeks. The intervention group showed a decrease of pain and an improvement of function, mood, balance, and flexibility. The CG showed no significant differences. In 2014, Albert Anand et al^48^ published a study with the aim of evaluating the benefits of modified Pilates method for patients with chronic nonspecific LBP. The sample was randomly divided into 2 groups; the subjects of group A underwent a modified specific Pilates method with flexibility exercises and the subjects of group B underwent therapeutic exercises with flexibility exercises. The experimentation was conducted for a period of 8 weeks. Through the adoption of the Oswestry Disability Index and the VAS,^55,56^ they found that the modified Pilates method works in reducing pain, improving back-specific function, improving general health (personal car and social life), and improving flexibility in individuals with nonspecific CLBP. Results were not so remarkable on group B. Another study of particular importance is the experimentation of Natour et al^53^; the authors analyzed 60 patients with nonspecific LBP. The sample was divided into 2 groups: the EG maintained medication treatment with the use of nonsteroidal anti-inflammatory drug (NSAID) and, in addition to, underwent treatment with the Pilates method, whereas the CG continued medication treatment with the use of NSAID and did not undergo any other intervention. An examiner blind to the assignment of the patients performed all evaluations at the following times: (T0) immediately prior to the study randomization (baseline); (T45) 45 days after T0; (T90) 90 days after T0 (conclusion of the Pilates method); and (T180) 90 days after the conclusion of the exercise program. Statistical differences favoring the Pilates group were found with regard to pain (VAS index, *P* < 0.001) and function (Roland-Morris questionnaire, *P* < 0.001). Statistical differences were also found between groups regarding the use of pain medication at 45, 90, and 180 days of exercise program (*P* < 0.010), with the Pilates group taking fewer NSAIDs than the CG that continued medication treatment with the use of NSAID and did not undergo any other intervention. Last, Notarnicola et al^54^ showed that 5 lessons per week for a period of 6 months of Pilates method is effective for the management of CLBP (*P* < 0.05) and that the inactivity contributes to further worsening, inducing a vicious cycle in which pain and physical activity intolerance follow each other.

#### Comparison of the Pilates Method With Other Exercise Programs for the Pain Outcome

Within this section, 6 studies^29,33,40,41,43,52^ were included in the analysis. In these studies, the pain was evaluated before and after intervention; in addition, the results were compared with CGs that underwent alternative exercises. In 2006, Donzelli et al^29^ enrolled 53 patients with at least 3 months of nonspecific LBP; the subjects were entered into a Pilates therapy or a back school treatment group, but only 43 subjects completed the study. After 6 months of treatments, a significant reduction in pain intensity (VAS score) and disability (the Oswestry disability index) was observed across the entire sample but the Pilates method group showed better compliance and subjective response to treatment. Curnow et al^33^ compared the effects of 3 different Pilates method regimes on chronic, mild LBP symptoms. All groups showed statistically significant reductions in frequency, intensity, and duration of LBP across the weeks of exercising but there were no significant differences between the groups relative to each other. Wajswelner et al^40^ compared the efficacy of Pilates method with general exercise for CLBP. The entire sample showed significant improvements. Similar results were found at the 12 and 24 weeks follow-up in both the groups. In 2013, Kucukcakir et al^41^ evaluated the effects of Pilates method on pain, functional status, and quality of life in women with postmenopausal osteoporosis. Patients were randomly allocated into 2 groups (home exercise and Pilates method groups). Patients in the Pilates method group underwent a supervised Pilates method twice a week for 1 year. Patients in the home exercise group were asked to perform a home exercise program consisting of thoracic extension exercises. Patients were evaluated at baseline and after 1 year of participation in the exercise programs. All the samples showed significant improvements but were significantly greater in the Pilates exercise group compared with the home exercise group in all parameters. Similarly, Lee et al,^52^ after 8 weeks of intervention, showed a pain's decrease in both the study groups (*P* < 0.05), the Pilates mat group and the Pilates apparatus exercise group, but the Pilates mat group showed a greater decrease than the Pilates apparatus exercise group (*P* < 0.05). Finally, Marshall et al^43^ confirmed that 8 weeks of specific Pilates method for trunk (64 patients with LBP) had reduced the disability and the pain significantly.

#### Assessment of the Possible/Potential Therapeutic Effect of the Pilates Method on CLBP in Randomized Cohorts

Five studies^31,42,49–51^ were analyzed within this section. The pain was evaluated before and after intervention, using scale measures for pain that were validated for the measurement and the comparison. In 2008, after 1-year period of Pilates method intervention on 59 patients, Lim et al^31^ suggested that the Pilates method has beneficial effect in reducing symptom of LBP. The authors found a clinically significant reduction in Oswestry disability index score (*P* < 0.001) and there was also an association between improvement in pain reduction and frequency of attendance (*r* = 0.314, *P* = 0.028). Similarly, Mallin and Murphy^42^ showed significant differences using the NRPS^57^ after 12 weeks of intervention (*P* < 0.01). However, after 6 weeks, the modifications were not confirmed (*P* > 0.05). In 2014, Borges et al^49^ studied a sample of 22 patients diagnosed with myelopathy/tropical spastic paraparesis caused by human T-lymphotropic virus type 1. LBP is the most common type of pain in these patients. Therefore, the Pilates method induced significant reduction in pain intensity (*P* < 0.001) and in almost all domains of the SF-36.^58^ Da Luz et al^50^ analyzed 86 subjects that were randomly allocated to 1 of the 2 groups: a Pilates mat group (n = 43) and an equipment-based Pilates group (n = 43); in this case, no benefits were observed for pain intensity in patients with chronic nonspecific LBP. In 2014, Kim et al^51^ showed a significant decrease of pain index (VAS) after 12-week of Prop Pilates Exercise Program.

### Analysis of Reviews

Nine studies were analyzed within this section.^12,14,32,35,36,38,39,44,47^ A close examination of reviews was conducted to critically evaluate and summarize the results of all published systematic reviews (with and without meta-analysis) that have investigated the effectiveness of Pilates method exercise in reducing pain and disability in people with CLBP. La Touche et al^12^ suggested that Pilates method reduces pain and disability, whereas Lim et al^35^ reported that Pilates method reduces pain when compared with minimal treatments, but not disability. In contrast, Pereira et al^38^ in 2012 concluded that Pilates method is ineffective in reducing pain and disability, and Posadzki et al^36^ in 2011 suggested that evidence was inconclusive. The above-mentioned studies adopted similar outcome measures for pain. On the contrary, Lim et al,^35^ Aladro-Gonzalvo et al,^14^ and Pereira et al,^38^ adopted different outcome measures for pain. In addiction, La Touche et al,^12^ Lim et al,^35^ and Pereira et al^38^ investigated people with nonspecific LBP. Interestingly, Posadzki et al^36^ included an additional primary study that included participants with LBP related to disc pathology in the lumbar spine.

In line with Posadzki et al,^36^ in 2013 Wells et al^47^ highlighted the insufficient number of studies and the poor methodological quality of available evidences; so, accordingly, they concluded that there was inconclusive evidence that Pilates method is effective in reducing pain and disability in people with CLBP. Moreover, Miyamoto et al^44^ and Sullivan et al^39^ asserted that Pilates method was better than a minimal intervention for reducing pain and disability in patients with CLBP but Pilates method was not better than other types of exercise for short-term pain reduction. However, the authors suggest that Pilates method can be recommended for the reduction of pain and disability, but no definitive conclusion can be made regarding the analyzed outcomes in the medium term.

## DISCUSSION

The Pilates method, using functional exercises, improves the muscular strength and endurance.^59^ While practicing, the level of these exercises increases week after week and consequently determines one important postural control improvement.^16^ In 2009, Curnow et al^33^ showed that the Pilates method improves load transfer through the pelvis. Previously, in 2005, Gagnon et al^60^ concluded that there is no significative difference between Pilates method and other exercises for lumbar stabilization. In 2013, Pereira et al^38^ confirmed Gagnon conclusions but, in addiction, in this case the authors stated that the principles of the Pilates method are similar compared with other generic lumbar exercises. Our systematic review explores the clinical effectiveness of Pilates method in patients with LPB through a critical review of the literature. Nevertheless, this review indicates that there is heterogeneity at various levels including methodology, physical examination, population, the intervention itself, and the outcome measures. The interesting outcome is that all the included articles focused on functional disability and pain. All of the studies opted to begin sessions with basic exercises, but the duration or frequency of sessions were significantly different. However, our systematic search shows evidence that Pilates method-based exercises are more effective than no treatment or minimal physical exercise interventions in the management of chronic nonspecific LBP. Our results, pointed out that the effects of the Pilates method are only proven for patients with chronic nonspecific LBP in the short term and it is still not possible to make inferences regarding the effects of treatment over time. Of interest, a recent study by Natour et al^53^ showed that the group of participants that were practicing Pilates method resulted statistically different compared with the CG regarding the use of pain medication at 45, 90 (conclusion of the Pilates method), and 180 days, 90 days after the conclusion of the exercise program (*P* < 0.01).

In conclusion, the level of “physical exercise,”^30^ the frequency, and the intensity/workload of Pilates protocols applied resulted vague and often undefined. Moreover, there is not homogeneity in terms of control and intervention group or intervention therapy in many studies analysed.^16,29,30^Table 1   clearly shows that there is a dearth of well-designed studies that clearly demonstrates the efficacy of a specific exercise program over another in the treatment of chronic pain. However, the consensus in the field suggests that Pilates method is more effective than minimal physical exercise intervention in reducing pain and disability in the short-term period. In other words, there is agreement that exercise “helps” in the treatment of chronic pain, but it is still not clear exactly which factors or particular kind of exercises may be responsible of such improvements. Further studies should be carried out in order to better understand the short-term and long-term effects of Pilates programs on LBP reduction.
